# Country-level factors dynamics and ABO/Rh blood groups contribution to COVID-19 mortality

**DOI:** 10.1038/s41598-021-04162-2

**Published:** 2021-12-31

**Authors:** Alfonso Monaco, Ester Pantaleo, Nicola Amoroso, Loredana Bellantuono, Alessandro Stella, Roberto Bellotti

**Affiliations:** 1 Istituto Nazionale di Fisica Nucleare (INFN), Sezione di Bari, Via A. Orabona 4, 70125 Bari, Italy; 2 Dipartimento di Scienze mediche di base, Neuroscienze e organi di senso, Piazza G. Cesare 11, 70124 Bari, Italy; 3 Dipartimento Interateneo di Fisica “M. Merlin”, Università degli Studi di Bari “‘Aldo Moro”, Via G. Amendola 173, 70125 Bari, Italy; 4 Dipartimento di Farmacia - Scienze del Farmaco, Università degli Studi di Bari “Aldo Moro”, Via A. Orabona 4, 70125 Bari, Italy; 5 Dipartimento di Scienze biomediche e oncologia umana, Università degli Studi di Bari “Aldo Moro”, Bari, Italy

**Keywords:** Computational biology and bioinformatics, Genetics, Immunology, Risk factors

## Abstract

The identification of factors associated to COVID-19 mortality is important to design effective containment measures and safeguard at-risk categories. In the last year, several investigations have tried to ascertain key features to predict the COVID-19 mortality tolls in relation to country-specific dynamics and population structure. Most studies focused on the first wave of the COVID-19 pandemic observed in the first half of 2020. Numerous studies have reported significant associations between COVID-19 mortality and relevant variables, for instance obesity, healthcare system indicators such as hospital beds density, and bacillus Calmette-Guerin immunization. In this work, we investigated the role of ABO/Rh blood groups at three different stages of the pandemic while accounting for demographic, economic, and health system related confounding factors. Using a machine learning approach, we found that the “B+” blood group frequency is an important factor at all stages of the pandemic, confirming previous findings that blood groups are linked to COVID-19 severity and fatal outcome.

## Introduction

The first information on a cluster of cases of “‘pneumonia of unknown cause” or “‘viral pneumonia” was notified to WHO’s country offices in the People’s Republic of China on December 31st 2019^1^. Since then, as of April 19th 2021, 141,642,813 global COVID-19 cases have been recorded with more than 3 million deaths^2^. While these numbers would lead to a rough estimate of the case fatality rate (CFR) of around 2.1% worldwide, wide differences are observed in country-specific death rates. As an example, in the same previously mentioned date of April 19th 2021 the CFR ranged from 9.21% in Mexico to 0.05% in Singapore^3^. The reason for this diversity in country specific CFRs has only recently been investigated. Among factors under scrutiny there were comorbidities such as obesity^4^, diabetes^4–6^, high blood pressure^7^, general indicators of the quality of healthcare systems including the number of hospital beds per thousands^8^ or the number of tests per thousands^9^, and the age population structure with specific reference to the percentage of residents aged >70 where a higher CFR has been generally observed^10^. The large majority of studies published so far, have investigated the contribution of these factors during the first wave of the COVID-19 pandemic. However, two further waves of cases with their load of casualties have been experienced since the release of these first reports. Further, large collaborative studies on COVID-19 cases enrolled during the first wave of the pandemic have identified the first set of genetic loci possibly responsible for the observed wide variation in symptoms severity^11–14^. After the initial outbreak in China, the most severely hit countries were those with a high gross domestic product (GDP) per capita and well-established healthcare systems. In contrast, COVID-19 casualties appeared to be lower in selected world regions such as the Middle East, South Eastern Asia and lower income countries. Several factors can explain the observed differences in the first wave of COVID-19, including but not limited to under-reporting of cases, inadequate testing, challenges in the attribution of the cause of death. Some studies have shown how the observed differences depend on rather intuitive factors such as the age structure of the country population, the prevalence of comorbidities and societal dynamics^15^. Other studies have considered less obvious factors: virus strains^16,17^, differences in genetic background^18–21^, bacillus Calmette-Guerin trained immunity^22–25^ and also air pollution^26–28^ or the political regime^29^. In this study, we aimed to investigate the role of ABO/Rh blood groups in the different waves of the pandemic and to assess how these factors’ contribution changed over time, while accounting for the main demographic, economic and health system related confounding factors. For this purpose we used an approach based on artificial intelligence which is capable of integrating the effects of several factors and their interactions in a multivariate nonlinear model. Using the total deaths per million caused by COVID-19 (TDPM) and indicators such as demographic, economic, health system related and genetic factors, this work provides a quantitative analysis of the SARS-CoV-2 pandemic in several countries through a longitudinal study sampled at three different times: June, September and December 2020. Next, we determined the specific contribution of different features to the pandemic severity. Noticeably, the B+ blood histotype became more important as pandemic progressed while “diabetes prevalence” and “cardiovascular death rate” lost importance. As far as we know, our study is one of the first that combines different kinds of features, genetic and non genetic, in a complex forecasting model that exploits the potential of machine learning to study different waves of the COVID-19 pandemic. The reported results show that factors contribution to the COVID-19 spreading could change over time and depend on the pandemic stage. While we acknowledge that additional variables are important to explain the large variability in TDPM across countries we nonetheless believe that our analysis can help in better understanding COVID-19, its spreading, and in developing effective measures to reduce its death toll.

## Methods

### Data

Our aim was to explore whether ABO and Rhesus blood group frequencies could predict the total deaths per million caused by COVID-19 (TDPM). We explored this relationship at three different time points, June 15 2020, September 15 2020, and December 15 2020, which cover the second half of 2020, i.e. the period corresponding to the end of the first pandemic wave, the beginning of the second outbreak, and the apex of this second stage. To build a prediction model we used a set of 10 indicators of ABO and Rhesus blood group frequencies, or “genetic” features^30^. In addition we included a set of 12 “non-genetic” features downloaded from OWID^31^ and updated to 2020 including demographic, economic, medical, and life style indicators. Table 1 contains a complete list of the input features used to build a predictive model of the TDPM. To get a wide and varied perspective we used data from 75 worldwide countries listed in Table 2 (35 European, 21 Asian, 7 African, 6 North American, 4 South American, 2 Oceanian countries). The study was carried out in accordance with the relevant national and international guidelines.

**Table 1:** List of input features.

Genetic features	Demographic indicators	Medical indicators	Economic indicators	Life style indicators
O+ A+ B+ AB+ O− A− B− AB− *O*/non *O* Rh−/Rh+	Population density	Life expectancy at birth	GDP per capita	Percentage of female smokers
	Median age of the population	Cardiovascular death rate	Total healthcare expenditure	Percentage of male smokers
	Population aged 65 or older	Diabetes prevalence	Hospital beds per thousand inhabitants	
	Population aged 70 or older			

We used five kinds of features: genetic, demographic, economic, medical, and life style indicators.

**Table 2:** List of input countries.

Countries		
Austria	Belgium	Bosnia and Herzegovina
Bulgaria	Cyprus	Croatia
Czechia	Denmark	Estonia
Finland	France	Germany
Greece	Hungary	Iceland
Ireland	Italy	Lithuania
Luxembourg	Malta	Moldova
Montenegro	Netherlands	Norway
Poland	Portugal	Romania
Russia	Serbia	Slovakia
Slovenia	Spain	Sweden
Ukraine	United Kingdom	Armenia
Bangladesh	Bahrain	China
India	Indonesia	Iran
Israel	Japan	Lebanon
Myanmar	Malaysia	Nepal
Philippines	Singapore	Saudi Arabia
South Korea	Thailand	Turkey
United Arab Emirates	Yemen	Ethiopia
Ghana	Kenya	Mauritius
Morocco	South Africa	Zimbabwe
Canada	Costa Rica	Dominican Republic
Jamaica	Mexico	United States
Brazil	Chile	Colombia
Ecuador	New Zealand	Australia

### Data analysis

**Figure 1:**
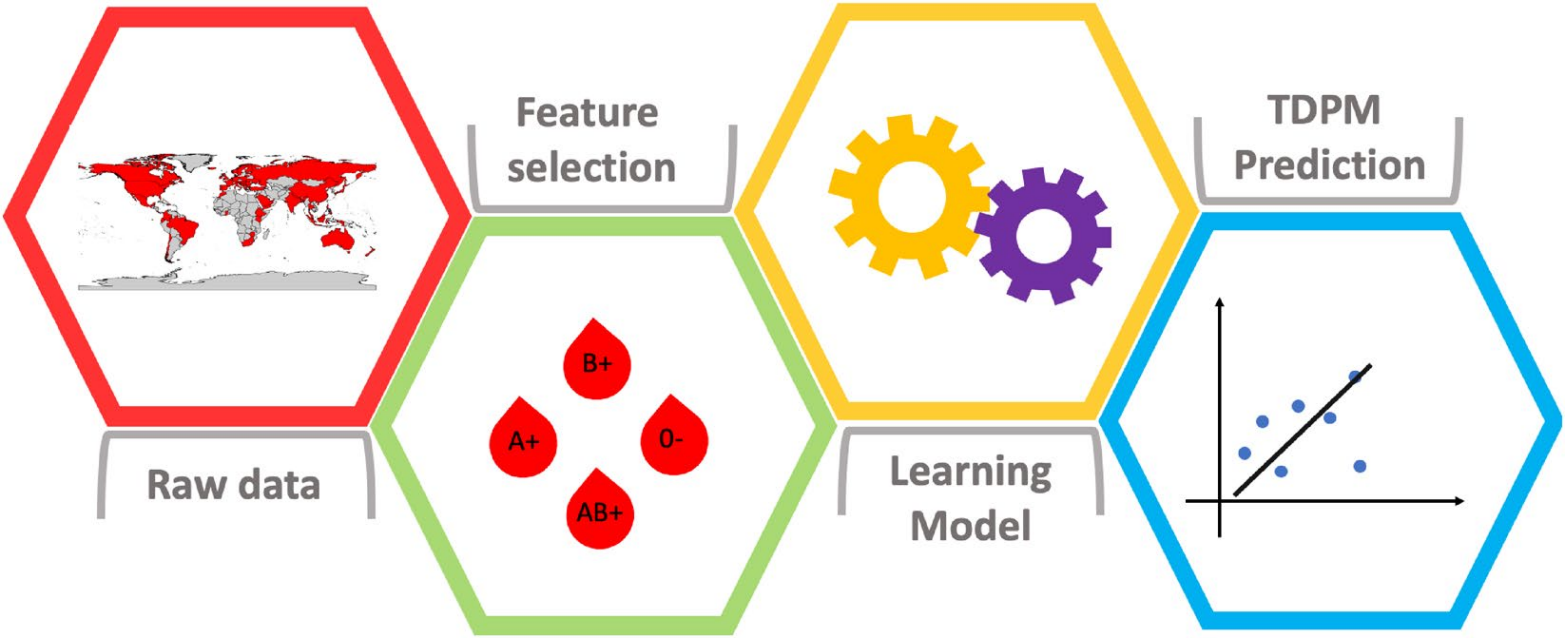
Flowchart of the proposed methodology. We fed the learning algorithm with selected features to forecast the TDPM. Microsoft Power Point was used to generate the figure.

**Figure 2:**
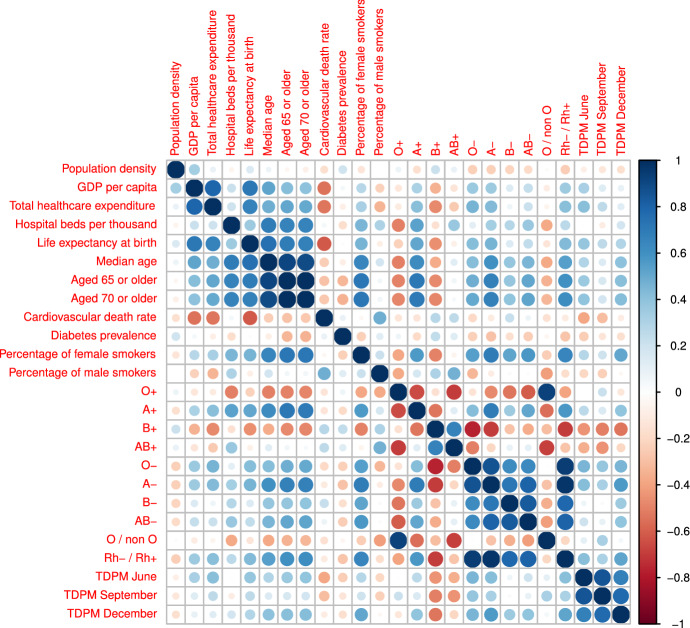
Correlation matrix of all variables (both dependent and independent). As expected “Median age of the population”, “Population aged 65 or older”, and “Population aged 70 or older” have mutual correlation close to 1; also the genetic features are highly correlated with each other. The TDPMs at the three different dates are also highly correlated with each other, as expected. Notably, the “B+” predictor has the highest (and negative) linear correlation with the TDPM at all three dates. R package corrplot 0.90 was used to generate the figure.

For the prediction of the TDPM we used a machine learning approach based on a versatile and non linear machine learning algorithm, the Random Forest (RF) model. The data analysis procedure is summarized in Fig. 1. Given the relatively high mutual correlation of the chosen predictors (see Fig. 2), we fed the RF algorithm with a subset of the whole set of features. We used the Boruta wrapper to select relevant features. This is a typical and widely used scheme in machine learning analysis: first selecting the features that maximize model performances through a wrapper algorithm; then passing as input to the algorithm only the most informative features to reduce the noise. This approach minimizes the risk of incurring in typical machine learning problems such as overfitting and underfitting. The choice of the Random Forest algorithm in the first place is motivated by the same reason. In RFs individual decision trees are characterized by high variance and low bias, but by averaging over the variance of tree outputs, RFs have low bias and moderate variance^32^. To prove the robustness of our results we also tested a linear multivariate model for comparison. The results and plots presented in this article were obtained using R version 4.0.5.

### Feature selection

We performed feature selection using a robust and efficient algorithm called Boruta^33^, which is a wrapper method based on the Random Forest algorithm (described in the next section). Briefly, Boruta (Boruta is a god of the forest in the Slavic mythology) exploits the same idea that originates the Random Forest method: it perturbs the system with elements of randomness and computes results from the set of randomized samples, thus decreasing the negative upshot of random instabilities and correlations inherent in a classification or a forecasting algorithm.

During the training phase, Boruta enlarges independent Random Forest trees on different bagging samples building *shadow features*, or copies of the original features with shuffled values, and compares the importance of the original features with the importance of their random shuffled copies. In other word it uses a permutation procedure to validate the importance assigned to the features by the RF algorithm, increasing the robustness of the methodology: shadow attributes play the role of reference values for deciding which attributes are important. *Tentative features* have an importance that is so close to their best shadow features that Boruta cannot make a decision with the desired confidence. By design, Boruta selects all features which are relevant to the outcome variable prediction and the selected features yield a minimal forecasting error. Specifically, Boruta performs the following steps^34^:

- Permute each feature $X_j$ to generate a shadow feature $X_j^{(s)}$;
- Fit a Random Forest using both the original and the shadow features;
- Compute importance of each feature $X_j$ and $X_j^{(s)}$ by means of Mean Decrease Accuracy. Then compute a Z score from the ratio between the mean accuracy loss and the standard deviation of the same distribution;
- Find the maximum Z score among shadow attributes (MZSA);
- Call $X_j$ important for a single run if its Z score is higher than the Z score of MZSA;
- Apply a two-sided statistical test for all features with null hypothesis that the variable importance is equal to the maximal importance of the MZSA. For each feature $X_j$ the algorithm counts how many times, on *M* runs, the importance of $X_j$ is higher than MZSA (a hit is recorded for the variable). The expected number of hits, according to a binomial distribution with $p =$ $q =$ 0.5 is $E(M) = 0.5M$ with standard deviation $S = \sqrt{(}0.25M)$. Then $X_j$ is tagged as important when the number of hits is significantly higher than *E*(*M*), and tagged as unimportant when the number of hits is significantly lower than *E*(*M*).
- Repeat the previous steps for a predefined number of iterations, or until all attributes are tagged.

### Learning model

A Random Forest (RF) is composed by an ensemble of classification/regression trees made by means of bootstrapping of the training dataset^35^. To improve forecast accuracy, RF combines multiple weak models to produce a powerful ensemble. Due to a randomization process of the input variables in the training phase, the RF trees have low mutual correlation. In fact, in the building step of the trees, at each node a subset of features is randomly selected. Furthermore, RFs have some characteristics that make them ideal in many machine learning analyses. For instance, they are simple to tune as most of the times they only require modulation of two parameters: the number of trees *n* and *m* the number of features sampled to grow each leaf within a tree. Furthermore RF can evaluate the importance of each input feature during the training phase by means of the mean decrease of impurity, averaging over the whole forest of trees^35^. Moreover the RF algorithm is robust against overfitting and through an out-of-bag procedure it provides an unbiased estimate of the generalization error. Because it uses decision trees, the RF algorithm can capture non linear relationships with the input features.

In the present work we implemented a standard configuration in which each forest is composed by $n = 500$ trees and *m* is chosen to give the lowest RMSE. The optimal *m* is 2 at all time points.

### Cross validation and performance metrics

To increase the robustness of our procedure and minimize overfitting issues, we adopted a 5-fold classification framework. In other words, we divided the initial dataset of 75 nations into 5 random subsets without repetition. We used the union of 5 minus 1 subsets as training set and the remaining set for validation and repeated this procedure five times, which gave us five different training and validation sets, and therefore five models with their respective performances. The average of these five performance values is a reliable indicator of the overall model performance.

We measured performances in terms of coefficient of determination between predicted and actual values ($\hbox {R}^2$). In addition we evaluated the root mean square error (RMSE):

1 $$\begin{aligned} RMSE =\sqrt{\frac{1}{N} \sum _{i=i}^{N} \left( A_t - F_t\right) ^2} \end{aligned}$$

and the mean absolute error (MAE):

2 $$\begin{aligned} MAE =\frac{1}{N} \sum _{i=i}^{N} \left| A_t - F_t\right| \end{aligned}$$

where $A_t$ and $F_t$ are the actual and the forecast values, respectively. Both data processing and statistical analyses were performed in R version 3.6.1^36^.

## Results

We run the Boruta algorithm on the set of 22 input features to predict the TDPM at the three different time points. In all cases, many features assumed similar importance, and tentative and important features were difficult to discern as they were distributed similarly to shadow features (see Supplementary Fig. 1 in the Supplementary Information where shadow features are represented in blue, and tentative and important features in yellow and green, respectively). Noticeably, feature “B+”, or frequency of blood group “B+”, unambiguously stood out as the most important feature, well above other features in June, September and December.

**Figure 3:**
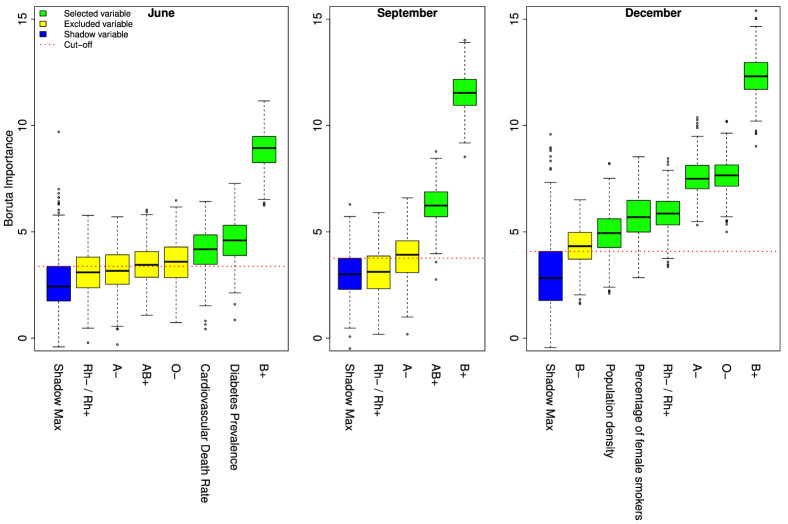
Boxplot of the distribution of the Boruta importance measure for input variables with median higher than variable “Shadow Max”. The distribution was obtained from 500 runs of the algorithm on the complete set of features using June, September, and December 2020 TDPM data. Using as cut-off the upper quartile of “Shadow Max”, we colored in yellow excluded variables and in green variables selected for further analysis. R base package graphics 4.0.5 was used to generate the figure.

Given the random nature of the Boruta algorithm, to establish which features to select as important among similarly important/tentative features, we run it 500 times with different random seeds and computed the distribution of the Boruta importance measure. At each time point we selected features using a cut-off criterion as follows. First, we excluded features having median of the Z score distribution below the median of the Z score distribution of the MZSA variable called “Shadow Max” in Fig. 3. Then, among the remaining features, we selected only features whose lower quartile was bigger that the upper quartile of the “Shadow Max” variable (drawn in red in Fig. 3). Using this procedure, we selected frequency of “B+”, “Diabetes Prevalence”, and “Cardiovascular death rate” in June, “B+” and “AB+” in September (“AB+” was not selected in June because, despite having median importance higher than “Shadow Max”’s median, it did not satisfy the chosen criterion), and six features in December, namely “B+”, “O−”, “A−”, “Rh−/Rh+”, “Percentage of female smokers”, and “Population density”. With this procedure we overcome a limitation of the Boruta algorithm, i.e. that its output can depend on the value of the random seed. To verify the stability of the algorithm with respect to the set of important features selected, we also performed Boruta 100 times (with different random seeds) over the whole dataset and counted the number of times each feature was selected as important by Boruta. Results show that the selected features are stable (see Table 3).

**Table 3:** Given the random nature of the Boruta algorithm we performed 100 runs of this algorithm on the same dataset with different seeds, then counted how many times each feature was selected by Boruta and reported counts in this table. Column “Type” has value “g” and “n” for “genetic” and “non genetic” features, respectively.

Name	Type	Percentage of times selected in		
		June	September	December
B+	g	100	100	100
Diabetes prevalence	n	100	0	0
Cardiovascular death rate	n	98	20	0
O−	g	93	4	100
AB+	g	91	100	0
Rh−/Rh+	g	42	1	100
A−	g	25	49	100
Total healthcare expenditure	n	4	0	0
O+	g	0	2	0
Percentage of female smokers	n	0	0	100
Population density	n	0	0	100
B−	g	0	0	75
A+	g	0	0	1
GDP per capita	n	0	0	0
Hospital beds per thousand	n	0	0	0
Life expectancy at birth	n	0	0	0
Median age	n	0	0	0
Aged 65 or older	n	0	0	0
Aged 70 or older	n	0	0	0
Percentage of male smokers	n	0	0	0
AB−	g	0	0	0
*O*/non *O*	g	0	0	0

**Figure 4:**
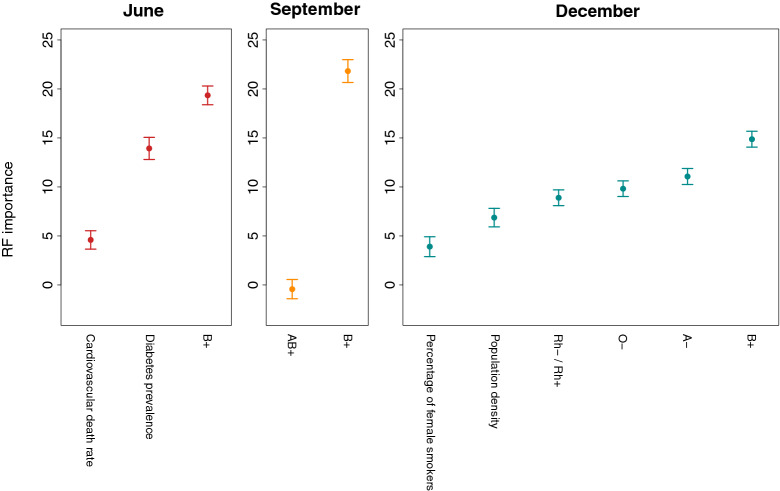
Average importance of the variables used in the RF model over 100 runs of the RF algorithm, with the respective standard deviations. R base package graphics 4.0.5 was used to generate the figure.

We then evaluated the RF regression model with the selected features in terms of $\hbox {R}^2$, RMSE, and MAE with a 5-fold CV procedure. Results are shown in Table 4. Figure 4 shows the average importance, within the RF model, of each of the selected features with the respective error bars.

Furthermore we used the selected Boruta features as input to a multivariate linear model, to check that the RF improves upon the linear model by adding a level of complexity (compare Tables 4 and 5), and to compare significant features. According to a Kruskal-Wallis test^37^ performances metrics of two implemented methods (specially $R^2$ and RMSE) are significantly different (*p*-value $< 1\%$).

Also the linear model finds “B+” important, at a 0.01 significance level, but doesn’t find the other features significant except for “Cardiovascular death rate” in June, while the overall multivariate linear model is highly significant with an R-squared ranging between 0.29 and 0.32 (see Table 5).

**Table 4:** Performance measures of the RF regression model at each selected time point, using the selected Boruta features and averaged over 5 runs of cross validation (with the respective standard deviations).

Time point	$\hbox {R}^2$	RMSE	MAE
June	0.47 ± 0.13	135 ± 10	85 ± 11
September	0.25 ± 0.19	192 ± 37	129 ± 24
December	0.34 ± 0.04	312 ± 48	241 ± 39

**Table 5:** Performance metrics of a linear multivariate model applied to the set of features selected by Boruta at each time point, using all countries, averaged over 5 runs of cross validation (with the respective standard deviations). The last column reports only significant features. Significance codes: ‘***’ 0.001, ‘**’ 0.01, and ‘*’ 0.05. The multivariate linear model found feature “B+” to be significant at all three time points, and also found “Cardiovascular death rate” to be significant but only in June. The significance of these features is higher in June and lower but similar in September and December, however most of the linearity is explained by the intercept of the linear model.

Time point	$\hbox {R}^2$	RMSE	MAE	significant features
June	0.31 ± 0.10***	138 ± 49	105 ± 36	B+ **, Cardiovascular death rate **
September	0.32 ± 0.15***	184 ± 36	149 ± 34	B+ **
December	0.29 ± 0.17***	329 ± 64	260 ± 44	B+ *

## Discussion

In the first wave of the COVID-19 pandemic, striking differences were reported in the case fatality rate of different countries. While many factors can confound the identification of potential determinants of the death rates caused by COVID-19, several studies have been released in the past months addressing the contribution of different elements to the wide variability in country-specific CFRs.

We decided to investigate the number of fatalities due to COVID-19 in relation to the entire population of analyzed countries (i.e., the total deaths/1 million population, TDPM) rather than the more frequently used case fatality ratio (i.e., mortality, CFR). These two parameters are influenced in different ways by multiple variables such as the number and type of diagnostic tests performed in each country or the modalities used to impute deaths to COVID-19. Since the CFR strictly relies on the number of tests performed in each country, and testing has not been homogeneously performed in different countries, we decided to focus on the TDPM parameter.

**Figure 5:**
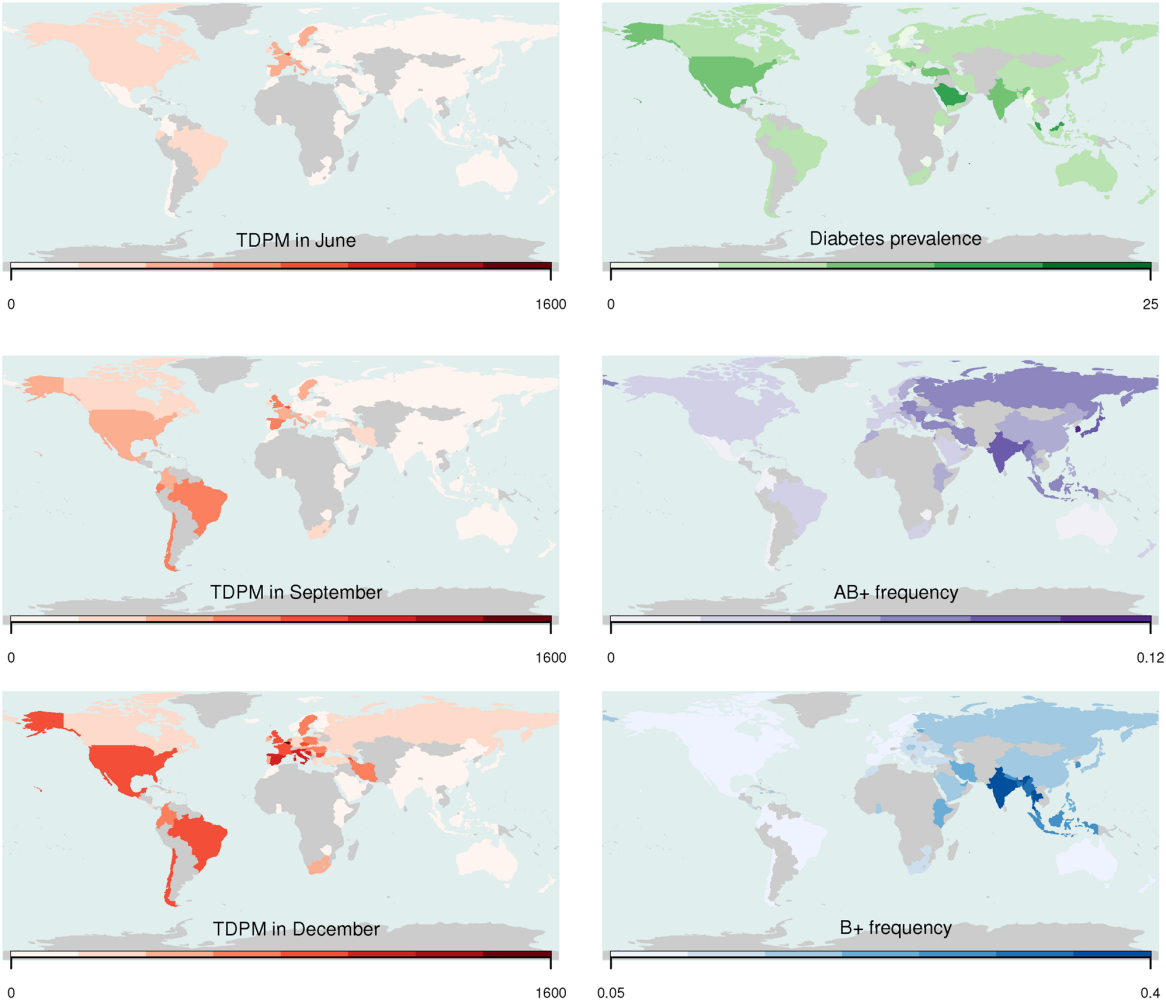
Map of the TDPM in June, September, and December 2020 on the left. Maps of some of the input features on the right. Countries not included in the analysis are colored in gray. R package Rworldmap 1.36 was used to generate the maps.

Features selected as input in our model were a combination of demographic, health and economic indicators, and frequencies of ABO and Rh blood groups. We chose ABO and Rh since several reports have indicated that both blood groups could influence the probability to progress to severe COVID-19 disease in SARS-CoV-2 infected subjects^18,38–42^. Further, while additional genetic loci have been identified in genome wide association studies, ABO and Rh blood groups offer the advantage of having frequencies available for almost all countries in the world. Figure 5 shows maps of TDPM at the three considered time points and the worldwide distribution of selected blood group frequencies.

In our study we explored the relationship between the total number of deaths per million caused by COVID-19 (TDPM), assessed in three different stages of the pandemic, a set of 12 selected country-level determinants and ABO and Rh blood group frequencies. The factors we analyzed do not represent an exhaustive collection of all possible variables that may play a role in the pandemics spreading but were selected for their relevance and diversity among the available variables in one of the largest, free, and daily updated databases on COVID-19^31^. Previous reports have analyzed the correlation of several variables with national case fatality rates but were limited to the first outbreak of COVID-19 (first half of 2020). To investigate putative predictors of TDPM, we used a non linear machine learning model combined with a feature selection procedure. In particular we implemented a typical machine learning framework based on the Boruta wrapper method to select only important features; a machine learning algorithm to forecast the TDPM; a cross validation procedure to make our results more robust. Furthermore, by running Boruta several times, changing the seed of the random generator at each run, we have obtained a more stable and reliable set of important features. We chose the Boruta feature selection method because the features of our model are highly correlated (see Fig. 2). A naive feature selection algorithm, for instance an algorithm that only keeps the ’minimal-optimal’ set of features, would discard one of two correlated variables. Boruta instead throws out only attributes that have no value to the classifier and keeps the ’all-relevant’ set of attributes. Our model shows that the frequency of “B+” in the population is an important predictor of the TDPM in June, September, and December 2020. A multivariate linear model confirmed the significance of the “B+” frequency predictor as protective against death by COVID-19. The “B+” is protective because it is negatively correlated with the TDPM as displayed in Fig. 2. RF outperformed the linear model as it can be deduced comparing Tables 4 and 5, which proves the existence of a complex (and not just linear) relationship between the input features and the outcome variable. Other factors emerged also as important to predict the TDPM although the model found them less important than frequency of “B+” and also their ability to predict the TDPM was not consistent over time: “Diabetes prevalence” and “Cardiovascular death rate” were important in June but not in September and December, in September frequency of “A−” switched from being tentative to being important, and stayed important in December together with “O−”, the ratio “Rh−/Rh+”, “Percentage of female smokers”, and “Population density” .

Interestingly, as the pandemic progressed, the number of important features predicted by Boruta grew to 6 in December (Fig. 3). Once more, the “B+” blood type frequency was the only feature always present, “A−” frequency was present twice, while no other feature had multiple occurrences. Thus, the putative role of ABO and Rh blood groups as determinants of countries TDPM seems to become more important with the progression of the pandemic (Fig. 4).

The ABO gene locus encodes for a protein responsible for the different ABO blood types. In fact, functional A and B alleles at the ABO genetic locus express A or B transferases (AT and BT respectively) which are able to add a different glycosyl group to the H antigene. The O allele lacks this enzymatic activity due to a truncating mutation. Very recently, the ABO plasma protein levels have been associated with COVID-19 susceptibility and severity^43^. Worthy of note, with a single exception, the genome-wide association studies published so far have been all concordant in indicating significant association with the ABO locus on chromosome 9 (9q34)^18,19,38,43–45^.

However, it is still unclear how the ABO protein modifies the COVID-19 risk. One hypothesis stems from in vitro experimental evidences showing that the interaction between the ACE2 protein and the SARS-CoV spike protein was inhibited by anti-A antibodies when the S protein was produced in cells capable of synthesizing the A blood group antigen^46^. More recently, the SARS-CoV-2 spike protein expressed in respiratory cells has been demonstrated to be specifically tagged with the corresponding A, B or H glycan epitopes of host cells^47^. Thus, the possibility that anti-ABO antibodies could play a role in protecting from infection and reducing the viral load is valuable and should not be overlooked. While in most publications the O blood group appears to confer a lower risk of COVID-19 compared to non-O blood groups (reviewed in^48^), the B blood group correlated with decreased risk of death in one of the first studies on the association between COVID-19 severity and blood type^42^, in a meta-analysis that systematically reviewed many studies on this topic^49^, and in a recent analysis of the association of ABO blood type with the early dynamics of the COVID-19 pandemic^50^. Also, it is worthwhile mentioning a recent study which documented that the ABO blood group-related histo-incompatibility might substantially reduce SARS-CoV-2 transmission. Importantly, the risk associated with a specific blood type changed, at population levels, depending on the epidemic phase (late vs early) and on the heterogeneity of blood type composition within specific populations or communities^51^. A report published while this work was under review, emphasizes the importance of blood histo-incompatibility demonstrating, in six different geographic regions, the dependency of the infection rate from country-specific blood groups distribution^52^. Hence, the results obtained in this work are in line with those described in recent studies with different methodologies and datasets. A second possible link between ABO and COVID-19 severity can be found in several studies documenting association of ABO blood groups with thromboembolic disease^53^. The intragenic *rs*505922 SNP has been shown to be responsible for differential ABO protein levels with an increasing effect for allele “C” and diminishing levels for allele “T”^54,55^. *rs*505922 is in LD with the O blood group SNP *rs*8176719, which has been repeatedly associated with an increased risk of venous thromboembolism^56–59^. The *rs*8176719 polymorphism has been also associated to Factor VIII levels^60^, malaria^61,62^, venous thromboembolism^63^, vWF levels^60^. In this scenario the ABO antigens would modulate the intravascular disseminated coagulation and endothelial dysfunction that earmark the severe form of COVID-19.

Similarly, recent findings suggest that the Rh blood group might be associated with severity of COVID-19, with “Rh−” having a protective role^41,42^. The increasing predominance of B+ as negatively associated to COVID-19 deaths, was paralleled by the disappearing of factors such as “diabetes prevalence” or “cardiovascular death rate”. Thus, it might be possible that in the initial phase of the pandemic, severe COVID-19 was targeting categories with favoring comorbidities. In later waves of the pandemic, with increasing acquired immunization possibly protecting this group of subjects, individual genetics could have played a larger role in the fatal outcome of COVID-19. Further, recent work reported that both B and Rh+ were protective against influenza due to identified zoonotic or pandemic influenza virus^64^. B was also protective against pneumonia due to S. pneumoniae. Also, the B blood group has a decreasing gradient of frequency from East Asia going westward. Thus, it is possible that the B allele has been under positive selection pressure by an ancient viral epidemic, which shaped the ancestral eastern Asian genome^65^.

This study has some limitations. We limited our analyses to determinants included in the OWID series, hence it is possible that additional factors not analyzed in our study might contribute to the TDPM differences observed between countries. As an example, vaccination against Bacillus Calmette-Guerin (BCG) has been recently reported as protective against severe COVID-19 infection^22,66–68^. However, a recent work has reported that, similarly with several determinants we investigated, BCG vaccination exerted a strong protective effect against COVID-19 in the early stage of the pandemic while fading in later stages^23^. Finally, we investigated only the ABO and Rhesus blood groups amongst many genetic loci that have been recently identified in genome wide association studies (GWAS)^14,19^, or more focused approaches^11,12,14,38^. However, ABO and Rh are part of a very limited group of genetic loci for which frequencies of the different phenotypic classes are available for almost all countries in the world. For the vast majority of SNPs only ethnic-specific frequencies can be extracted from available databases. In conclusion, differently from previous studies, in our investigation, the influence of genetic and non-genetic factors on the TDPM has been evaluated in different stages of the COVID-19 pandemic. Our findings suggest that in more advanced stages of the pandemic, individual genetic factors, and specifically the overall distribution of ABO and Rh blood groups distribution in the specific population, might exert a stronger influence on COVID-19 transmissibility and severity.

## Supplementary Information

Supplementary Information.

## Data Availability

The source code and data used to produce the results and analyses presented in this manuscript are available from Git repository https://github.com/esterpantaleo/covid_mortality.
