# Supplementary material for: Country-level factors dynamics and ABO/Rh blood groups contribution to COVID-19 mortality
Source: Sci Rep. 2021 Dec 31;11:24527. doi: 10.1038/s41598-021-04162-2 (PMC8720090; doi:10.1038/s41598-021-04162-2)
Supplement: Supplementary file 1 — Supplementary Information. [file 41598_2021_4162_MOESM1_ESM.pdf]

Supplementary Information for: Country-level factors dynamic and ABO/Rh blood groups contribution to COVID-19 mortality

Alfonso Monaco[1]¶, Ester Pantaleo[2]¶, Nicola Amoroso[1,3], Loredana Bellantuono[2], Alessandro Stella[4*], Roberto Bellotti[1,5]

1 Istituto Nazionale di Fisica Nucleare (INFN), Sezione di Bari, Via A. Orabona 4, 70125 Bari, Italy
2 Dipartimento di Scienze mediche di base, Neuroscienze e organi di senso, Piazza G. Cesare 11, 70124 Bari, Italy
3 Dipartimento di Farmacia - Scienze del Farmaco, Università degli Studi di Bari "Aldo Moro", Via A. Orabona 4, 70125 Bari, Italy
4 Dipartimento di Scienze biomediche e oncologia umana, Università degli Studi di Bari "Aldo Moro", Bari, Italy
5 Dipartimento Interateneo di Fisica "M. Merlin", Università degli Studi di Bari "Aldo Moro", Via G. Amendola 173, 70125 Bari, Italy

¶These authors contributed equally to this work.
* Corresponding author
Email: alessandro.stella@uniba.it

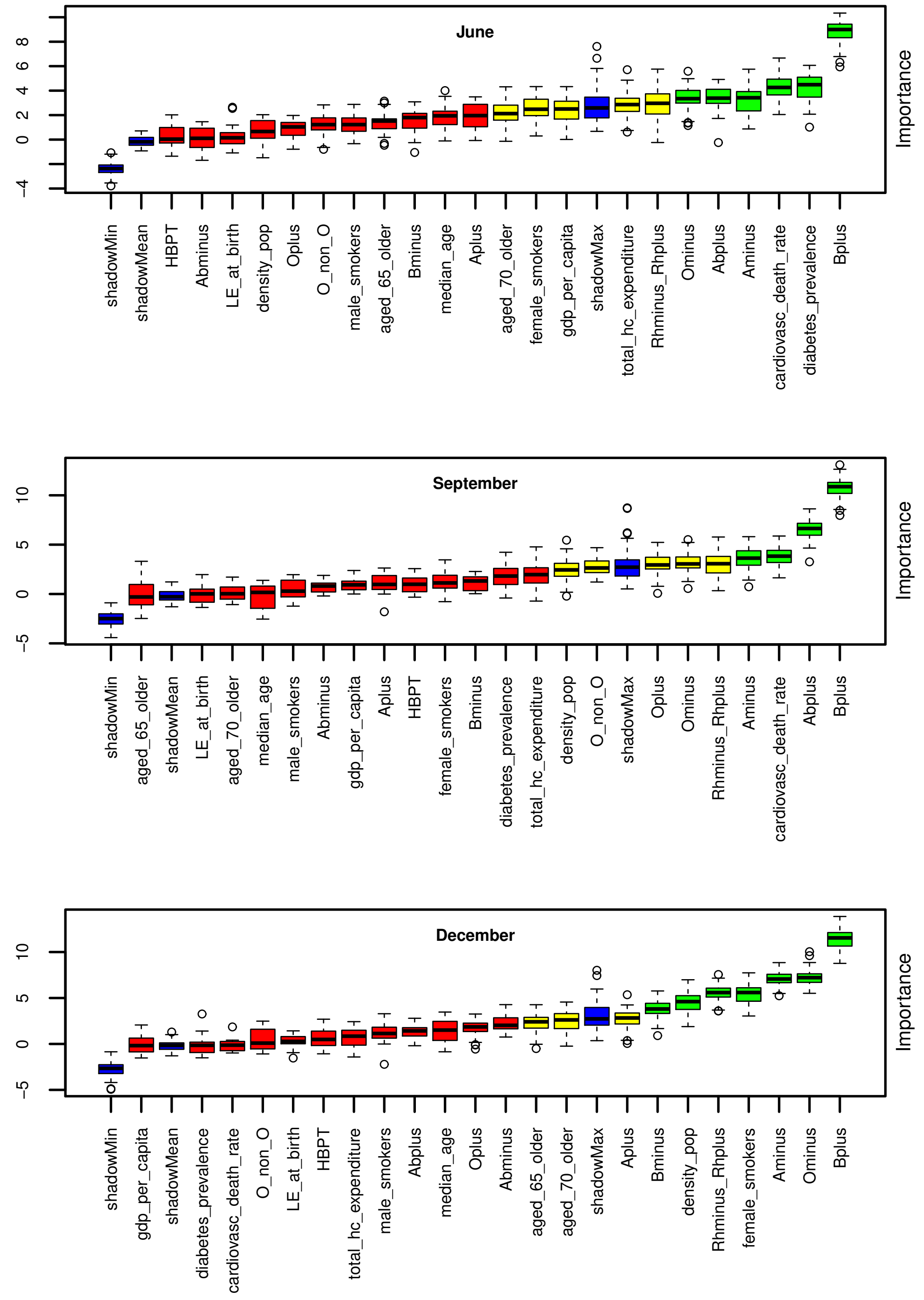

Supplementary Figure 1. Boxplot of the importance of each predictor, including shadow Boruta predictors (in blue) for the three time points in June, September, and December. Green colors indicate important features, red colors indicate unimportant features, and yellow colors tentative attributes, i.e., features that are almost as important as their best shadow attributes and Boruta cannot make a decision about them with the desired confidence.
